# DDX21 Promotes PCV3 Replication by Binding to Cap Protein and Inhibiting Interferon Responses

**DOI:** 10.3390/v17020166

**Published:** 2025-01-24

**Authors:** Haoyu Sun, Qianhong Dai, Beiyi Zhou, Xiaoyuan Lan, Yonghui Qiu, Qianqian Zhang, Dedong Wang, Yongqiu Cui, Jinshuo Guo, Lei Hou, Jue Liu, Jianwei Zhou

**Affiliations:** 1 College of Veterinary Medicine, Yangzhou University, Yangzhou 225009, China; sunhaoyu0222@163.com (H.S.); dqianhong@163.com (Q.D.); by214zhou000@163.com (B.Z.); lanxiaoyuan15@163.com (X.L.); 18238776375@163.com (Y.Q.); 15978357750@163.com (Q.Z.); wddyzu@163.com (D.W.); cuiyongqiu97@163.com (Y.C.); 15930270598@163.com (J.G.); hlbj09@163.com (L.H.); liujue@263.net (J.L.); 2 Jiangsu Co-Innovation Center for Prevention and Control of Important Animal Infectious Diseases and Zoonoses, Yangzhou University, Yangzhou 225009, China

**Keywords:** porcine circovirus type 3, capsid protein, DEAD-box RNA helicase 21, viral replication

## Abstract

Porcine circovirus type 3 (PCV3) is an emerging pathogen that causes porcine dermatitis, nephropathy syndrome-like symptoms, multisystemic inflammation, and reproductive failure. The PCV3 capsid (Cap) protein interacts with DDX21, which functions mainly through controlling interferon (IFN)-β levels. However, how the interaction between DDX21 and PCV3 Cap regulates viral replication remains unknown. In the present study, upon shRNA-mediated *DDX21* depletion in PK-15 cells, we observed impaired PCV3 proliferation via a lentivirus-delivered system, as indicated by reduced replicase (Rep) protein levels and viral titers. Furthermore, DDX21 negatively regulated IFN-β and interferon-stimulated gene (ISG) levels, promoting PCV3 replication. Mechanistically, PCV3 Cap co-localized and interacted with DDX21, and the nuclear localization signal (NLS) of PCV3 Cap and ^763^GSRSNRFQNK^772^ at the C-terminal domain (CTD) of DDX21 were indispensable to the interaction. Moreover, PCV3 infection prevented the repression of DDX21 to facilitate its pro-viral activity. Taken together, these results show that DDX21 promotes PCV3 replication by binding to the PCV3 Cap protein and prohibiting IFN-β response, which provides important insight on the prevention and control of PCV3 infection.

## 1. Introduction

Porcine circovirus type 3 (PCV3) is an emerging pathogen found in sows with porcine dermatitis and nephropathy syndrome (PDNS) [1]. PCV3 has been spread worldwide, causing huge economic losses [2,3]. There exists about fifty years of history for PCV3 infection [4]. In 2019, a PDNS-like disease was found in pigs infected with PCV3, showing that PCV3 is pathogenic and induces PDNS-like clinical symptoms [5]. Several studies have proven how host proteins binding to PCV3 Cap facilitates viral replication [6,7]. For example, PCV3 enters into PK-15 cells via clathrin- and dynamin-2-mediated endocytosis [8]. Nucleolar nucleophosmin-1 promotes viral replication by interacting with PCV3 Cap protein [9]. Heme oxygenase-1, carbon monoxide, and biliverdin inhibit PCV3 replication [10]. Ring finger protein 2 prohibits PCV3 replication by the degradation of its Cap protein [11]. However, the role of PCV3 Cap protein in the viral life cycle has only been partially demonstrated [12,13,14,15,16].

DEAD-box helicase 21 (DDX21) is a well-known member of helicase superfamily 2 and contains the conserved motifs responsible for its helicase activity. DDX21 possesses a FRGQR repeat involved in RNA binding and folding activities [17] and can bind directly to different types of RNA and promote its metabolism [18]. In addition, DDX21 unwinds RNA/DNA strands with the help of different functional domains [19]. Several reports have shown that DDX21 positively regulates innate immune responses. Briefly, DDX1 interacts with dsRNA via its helicase domain and binds to DDX21 to bridge the binding of DDX1 with DHX36. The knockdown of DDX21 decreases the poly (I: C)-induced IFN-I expression [20]. DDX21 also negatively regulates innate immunity [21,22]. DDX21 may balance innate immune signaling through inhibiting IFN-β production [23,24]. The cleavage of DDX21 reduces IFN-β expression through dampening the formation of the ternary complex [25]. In addition, DDX21 increases IFN-β promoter activation during dengue virus infection [26]. It is undoubtedly controversial that DDX21 antagonizes or induces IFN-I expression. PCV3 Cap protein has been found to interact with DDX21 [27], but whether the interaction between DDX21 and PCV3 Cap modulates innate immune responses and viral replication remains obscure.

Herein, we demonstrate that DDX21 expression promotes PCV3 replication. We further show that DDX21 inhibits IFN-β and interferon-stimulated gene (ISGs) expression, promoting PCV3 replication. PCV3 infection prevented the repression of DDX21 to facilitate its pro-viral activity. Mechanistically, PCV3 Cap co-localized and interacted with DDX21, and the PCV3 Cap NLS and ^763^GSRSNRFQNK^772^ of DDX21 were indispensable to the interaction. Taken together, these results will provide novel insights into the prevention and control of PCV3 infection.

## 2. Materials and Methods

### 2.1. Cells and Virus

The porcine kidney epithelial cell line PK-15 (CCL-33, ATCC, Manassas, VA, USA) was maintained in a minimal essential medium (Gibco, Thermo Fisher Scientific, Waltham, MA, USA). The porcine pulmonary alveolar macrophage 3D4/21 (ATCC) cells were cultured in Roswell Park Memorial Institute (RPMI) 1640 medium (Gibco). Human embryonic kidney epithelial (HEK) 293 T cells (CRL-3216, ATCC) were cultured in Dulbecco’s modified Eagle’s medium (DMEM; Gibco). The 10% fetal bovine serum (FBS) (S711-001S; LONSERA, Shanghai Shuangru Biology Science & Technology Co., Ltd., Shuangru, Shanghai, China) was supplemented in the all the media. The PCV3 LY strain was propagated and stored in our laboratory [5].

### 2.2. Antibodies and Reagents

Anti-FLAG affinity resin (A2220) was acquired from Sigma-Aldrich (St. Louis, MO, USA). Anti-GFP (B-2, sc-9996) mouse mAb was acquired from Santa Cruz Biotechnology (Dallas, TX, USA). Mouse anti-β-actin mAb was purchased from Sangon Biotechnology (D191048, BBI Life Sciences Corp., Shanghai, China). Rabbit mAb against DDX21 (ab182156) was purchased from Abcam (Cambridge, MA, USA). Rabbit mAbs anti-p-TBK1 (S172) (ab109272), anti-TBK1 (ab40676), anti-IRF3 (ab68481) were purchased from Abcam. Rabbit pAb anti-p-IRF3 (S396) (AP0623) was purchased from ABclonal Technology. Pig anti-PCV3 Rep antibody was obtained from our laboratory. Rabbit anti-pig IgG was purchased from Bioss (bs-0309Rs, Beijing, China).

### 2.3. Plasmid Construction

The PCV3 Cap mutants and NLSs from different species were constructed as previously described [28]. The DDX21 variants (accession no. KX396051.1) were previously constructed and stored in our laboratory [29].

### 2.4. Sodium Dodecyl Sulfate–Polyacrylamide Gel Electrophoresis and Immunoblotting

The cell lysates were used for Western blotting as previously reported [30]. SDS-PAGE and Western blotting were carried out as reported in a previous study [29].

### 2.5. Confocal Microscopy, Co-IP, and GST Pull-Down

Confocal microscopy was carried out as previously reported [31]. Co-immunoprecipitation and glutathione S-transferase pull-down assays were carried out as reported in a previous study [32].

### 2.6. Quantitative Real-Time Reverse Transcription PCR

A two-step RT-PCR was performed to measure the mRNA levels of DDX21, IFN-β, MX1, MX2, OAS1, ISG15, and GAPDH in 3D4/21 cells. Primers are listed in Table 1. RNA was extracted using the TRIzol reagent, and a Vazyme cDNA Synthesis Kit (Vazyme Biotechnology, Nanjing, China) was used to create cDNA. The cDNA isolated from the samples was further examined using a LightCycler real-time PCR detection device (Roche) and an AceQTM Universal SYBR qPCR Master Mix Kit (Vazyme Biotechnology). The 20.0 μL total volume of the PCR reaction comprised 10.0 μL of AceQTM Universal SYBR qPCR Master Mix, 1 μL of cDNA, 8.2 μL of DNase/RNase-free H_2_O, and 0.4 μL of forward and reverse primers, each with a final concentration of 0.2 M.

### 2.7. Statistical Analysis

All results are presented as mean ± standard deviation (SD). Statistical analysis was performed using Student’s *t*-test. Statistical significance was set as ns *p* > 0.05, * *p* < 0.05.

## 3. Results

### 3.1. PCV3 Infection Prevents the Repression of DDX21 at Protein and mRNA Levels

**Figure 1:**
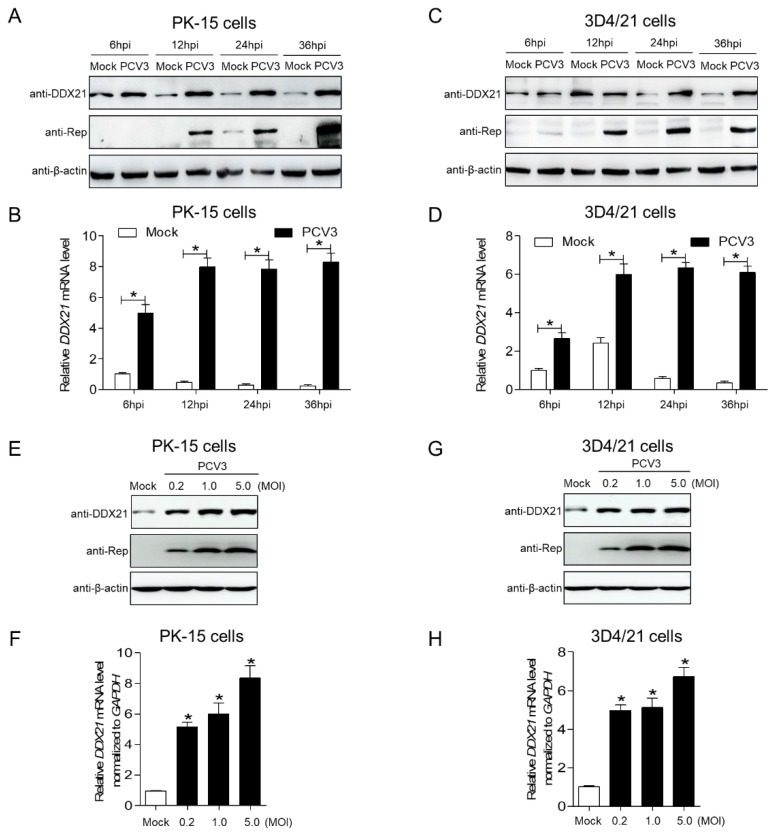
PCV3 infection prevents the repression of DDX21 at protein and mRNA levels. (**A**) PK-15 cells were inoculated with PCV3 at a MOI of 1.0 or mock-infected for 6, 12, 24, and 36 h and analyzed by immunoblotting with anti-DDX21, anti-Rep, and anti-β-actin antibodies, respectively. (**B**) qRT-PCR measurements of the *DDX21* mRNA at 6, 12, 24, and 36 hpi. (**C**,**D**) Proteins and DDX21 mRNA were extracted from PCV3-inoculated or mock-infected 3D4/21 cells at 6, 12, 24, and 36 h and analyzed by immunoblotting (**C**) and RT-qPCR (**D**), respectively. (**E**,**G**) PK-15 and 3D4/21 cells were inoculated with PCV3 at the indicated MOI of 0.2, 1.0, 5.0 or mock-infected for 24 h, and analyzed by immunoblotting respectively. (**F**,**H**) RT-qPCR measurements of the *DDX21* mRNA in PK-15 or 3D4/21 cells infected with PCV3 at the indicated MOI of 0.2, 1.0, 5.0 or mock-infected for 24 h. * *p* < 0.05.

In order to detect the DDX21 protein expression level upon PCV3 infection, we analyzed the kinetics of endogenous DDX21 expression in different cell types inoculated with PCV3, using Western blotting. The protein levels of DDX21 gradually decreased in the mock-infected PK-15 cells and fell to their lowest level at 36 h post-infection (hpi). However, the DDX21 protein levels in the PCV3-infected cells maintained higher levels than in the mock-infected cells (Figure 1A). Next, we wanted to determine whether the higher *DDX21* gene expression in the infected cells was caused by increased mRNA transcriptional levels; therefore, we performed quantitative reverse transcription-PCR (RT-qPCR) to detect the mRNA levels of *DDX21* at four time points with or without PCV3 infection. As shown in Figure 1B, the mRNA levels of *DDX21* were gradually reduced in the mock-infected cells. In the infected cells, the *DDX21* mRNA levels were much higher than in the mock-infected cells. Similar results were observed in the PCV3-inoculated 3D4/21 cells, except for 6 hpi (Figure 1C,D; *p* < 0.05), demonstrating that the DDX21 expression was repressed when the cells were infected with PCV3. The prevention of repression of DDX21 at the protein and mRNA levels occurred dose dependently in the PCV3-infected PK-15 cells (Figure 1E,F; *p* < 0.05) or in the 3D4/21 cells (Figure 1G,H; *p* < 0.05) at a multiplicity of infection (MOI) of 0.2, 1.0, and 5.0. The results showed that PCV3 infection prevents the repression of DDX21 at the protein and mRNA levels.

### 3.2. DDX21 Expression Promotes PCV3 Replication

**Figure 2:**
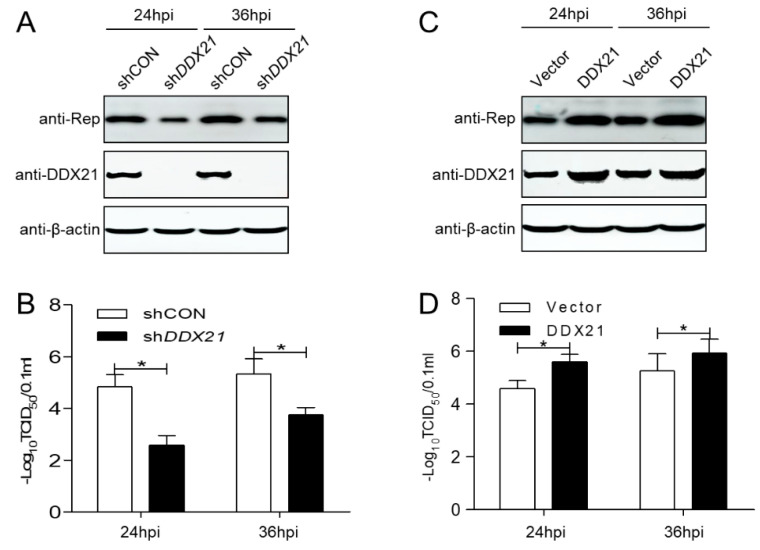
DDX21 expression facilitates PCV3 replication. (**A**,**C**) DDX21-overexpressing or *DDX21*-silenced PK-15 cells were inoculated with PCV3 at a MOI of 1.0 for 24, 36 hpi, and immunoblotting of Rep, DDX21, FLAG, and β-actin proteins. (**B**,**D**) TCID_50_ values of PCV3 in samples from (**A**,**C**). Viral stocks were harvested at 24, 36 hpi, and shCON-transfected or empty vector-transfected cells served as negative controls. Data are presented as mean ± SD from three independent biological experiments. * *p* < 0.05.

In order to investigate the function of DDX21 during PCV3 infection, the *DDX21*-silenced cells were infected with PCV3 and determined the Rep protein levels and the virus titer to detect viral replicative ability. Compared with the short hairpin control (shCON) group, PCV3 Rep protein expression in the *DDX21*-silenced cells was significantly reduced (Figure 2A). In addition, the viral titer of PCV3 was also reduced significantly in the *DDX21*-silenced cells (Figure 2B; *p* < 0.05). The data show that PCV3 replication is downregulated in *DDX21*-silenced cells.

Since *DDX21* silencing decreased PCV3 replication, we attempted to determine whether the overexpression of DDX21 facilitates PCV3 replication. Compared with the empty vector group, DDX21 overexpression increased PCV3 Rep protein (Figure 2C). Additionally, DDX21 overexpression upregulated PCV3 viral progeny production (Figure 2D; *p* < 0.05), suggesting that DDX21 overexpression upregulated PCV3 replication.

### 3.3. PCV3 Cap Co-Localizes and Interacts Directly with DDX21

**Figure 3:**
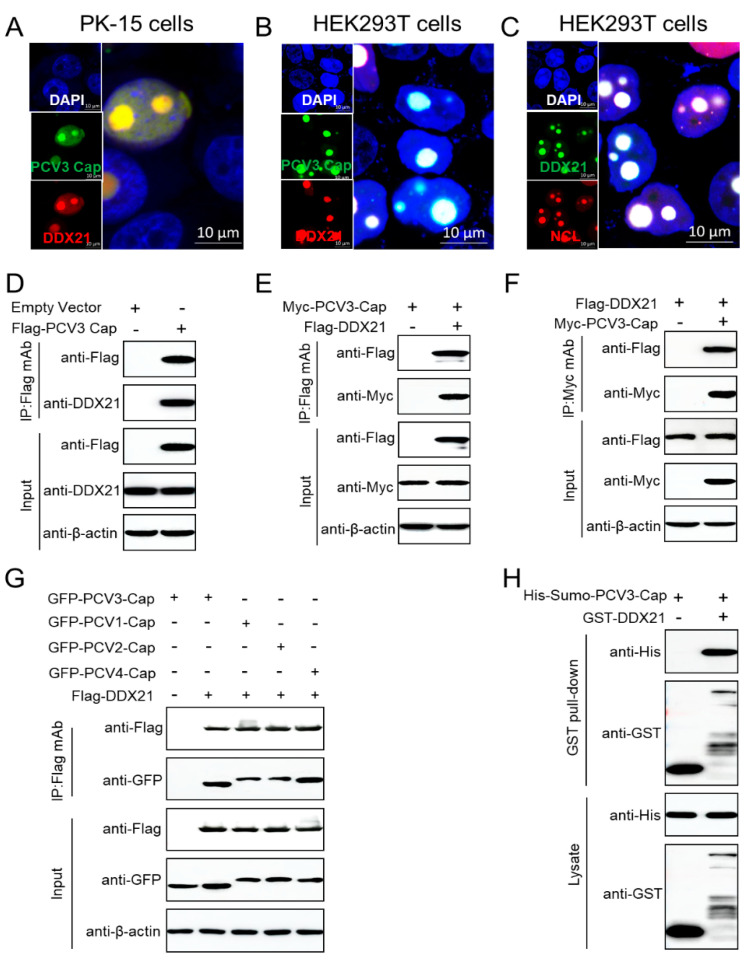
PCV3 Cap colocalizes and directly interacts with DDX21. (**A**) The co-localization of the viral protein Cap with DDX21. PK-15 cells were co-transfected with GFP-DDX21 and mCherry-PCV3-Cap for 24 h. (**B**,**C**) The co-localization of the viral protein DDX21 with PCV3 Cap or NCL. HEK293T cells were co-transfected with GFP-DDX21 and mCherry-PCV3-Cap (**B**) or mCherry-NCL (**C**) for 24 h. Cells were stained with DAPI and then observed under a confocal microscope. (**D**) Immunoprecipitation analysis. PK-15 cells were transfected with FLAG-PCV3-Cap for 48 h. (**E**,**F**) HEK293T cells were co-transfected with FLAG-PCV3-Cap and GFP-DDX21 or FLAG-DDX21 and GFP-PCV3-Cap for 48 h, and the cell lysates were immunoprecipitated with FLAG beads and then analyzed by immunoblotting with anti-FLAG, anti-GFP, and anti-β-actin antibodies. (**G**) The PCV1, 2, 3, 4 Cap proteins were indispensable to interaction with DDX21. (**H**) Sumo-PCV3-Cap bound to DDX21. GST or GST-DDX21 proteins were immobilized on GST beads and incubated with His-Sumo-PCV3-Cap. The targeted proteins were examined using immunoblotting with anti-His and anti-GST antibodies, respectively.

We observed the intracellular distributions of DDX21 and PCV3 Cap using confocal microscopy to evaluate the relationship between PCV3 Cap and DDX21 during transfection. DDX21 co-localized with PCV3 Cap co-transfected with GFP-PCV3-Cap and mCherry-DDX21 in the PK-15 cells (Figure 3A), mCherry-DDX21 in the HEK293T cells (Figure 3B), or GFP-DDX21 with mCherry-nucleolin (NCL) in the HEK293T cells (Figure 3C). Since NCL is a nucleolus-localized protein [33], and DDX21 overlapped with PCV3 Cap or NCL. The immunofluorescence assays show that PCV3 Cap or DDX21 is also a nucleolus-localized protein. PK-15 cells were transfected with FLAG-PCV3-Cap and subjected to immunoprecipitation with anti-FLAG beads, and the existence of DDX21 was detected with the anti-DDX21 polyclonal antibody (Figure 3D). In order to further verify the binding, FLAG-DDX21 and Myc-PCV3-Cap were co-transfected into HEK293T cells and then subjected to reciprocal coimmunoprecipitation. DDX21 interacted with PCV3 Cap (Figure 3E,F). To investigate whether the Cap proteins of PCV1, 2, 4 also interact with DDX21, FLAG-DDX21 and GFP-PCV3-Cap, GFP-PCV1-Cap, GFP-PCV2-Cap, or GFP-PCV4-Cap were co-transfected and immunoprecipitated with FLAG beads. The findings indicated that the PCV1, 2, 4 Cap proteins bind to DDX21 (Figure 3G). In addition, His-Sumo-PCV3-Cap and GST or GST-DDX21 were used to perform GST pull-down experiments. The results demonstrated that the DDX21 protein bound directly to PCV3 Cap (Figure 3H). To sum up, the data prove that PCV3 Cap co-localizes and interacts directly with DDX21.

### 3.4. The NLS of PCV3 Cap Is Crucial for Binding to DDX21

**Figure 4:**
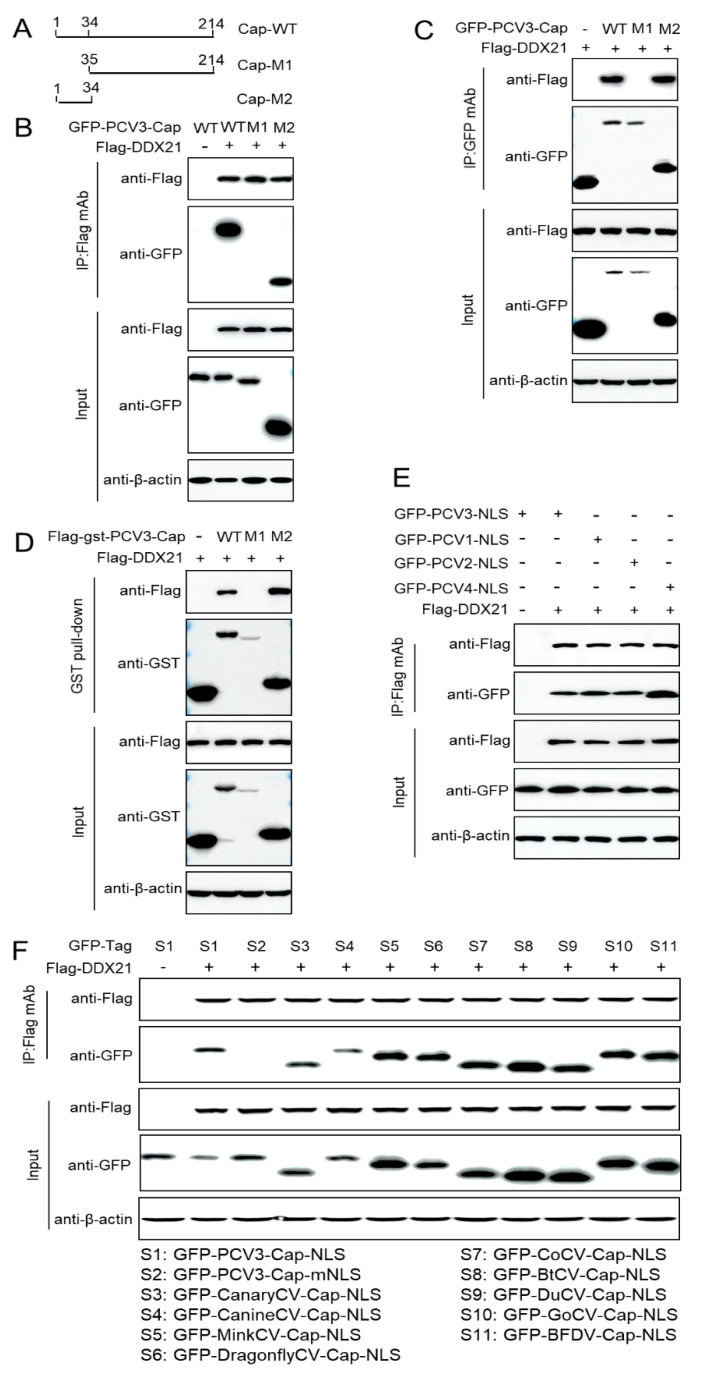
The PCV3 Cap NLS is crucial for binding to DDX21. (**A**) Schematic representation of the truncation mutants of PCV3 Cap used in this study. (**B**–**D**) The PCV3 Cap NLS interacted with DDX21. HEK293T cells were co-transfected with GFP-PCV3-Cap-WT, -PCV3-Cap-M1, -PCV3-Cap-M2 or FLAG-GST-PCV3-Cap-WT, -PCV3-Cap-M1, -PCV3-Cap-M2 with FLAG-DDX21, and cell lysates were then subjected to immunoprecipitation or GST pull-down assays. (**E**,**F**) HEK293T cells were co-transfected with Cap-NLSs within PCV1, 2, 3, 4 (**E**) and circoviruses from different species (**F**), along with FLAG-DDX21, and cell lysates were then subjected to immunoprecipitation assays.

The truncated PCV3 Cap variants were constructed to identify the PCV3 Cap binding domain responsible for its interaction with DDX21. The co-immunoprecipitation (Co-IP) assays show that PCV3 Cap-WT or PCV3 Cap-NLS-M2 interacted with DDX21 but PCV3 Cap-M1 did not (Figure 4A–C). The data show that PCV3 Cap-NLS was required for interaction with DDX21. Moreover, the GST pull-down experiments prove that PCV3 Cap-WT or Cap-NLS interacted with DDX21 but Cap-M1 did not (Figure 4D). These results further demonstrate that the PCV3 Cap NLS is the key domain for interaction with DDX21. To investigate whether the NLS within the capsid proteins of PCV1, 2, 3, or 4 interacted with DDX21, Co-IP experiments were performed and the results indicated that the NLS within the PCV1, 2, 3, or 4 Cap protein were required for interaction with DDX21 (Figure 4E). To further confirm the conserved characters of NLS in the interaction between Cap and DDX21, the results show that the Cap NLSs of circoviruses from terrestrial, aquatic, and avian species were essential for interaction with DDX21 as well (Figure 4F), demonstrating that the Cap NLSs of circoviruses from different species are all essential for the interaction with DDX21.

### 3.5. The DDX21-CTD Mediated Interaction with PCV3 Cap and Facilitated Viral Replication

**Figure 5:**
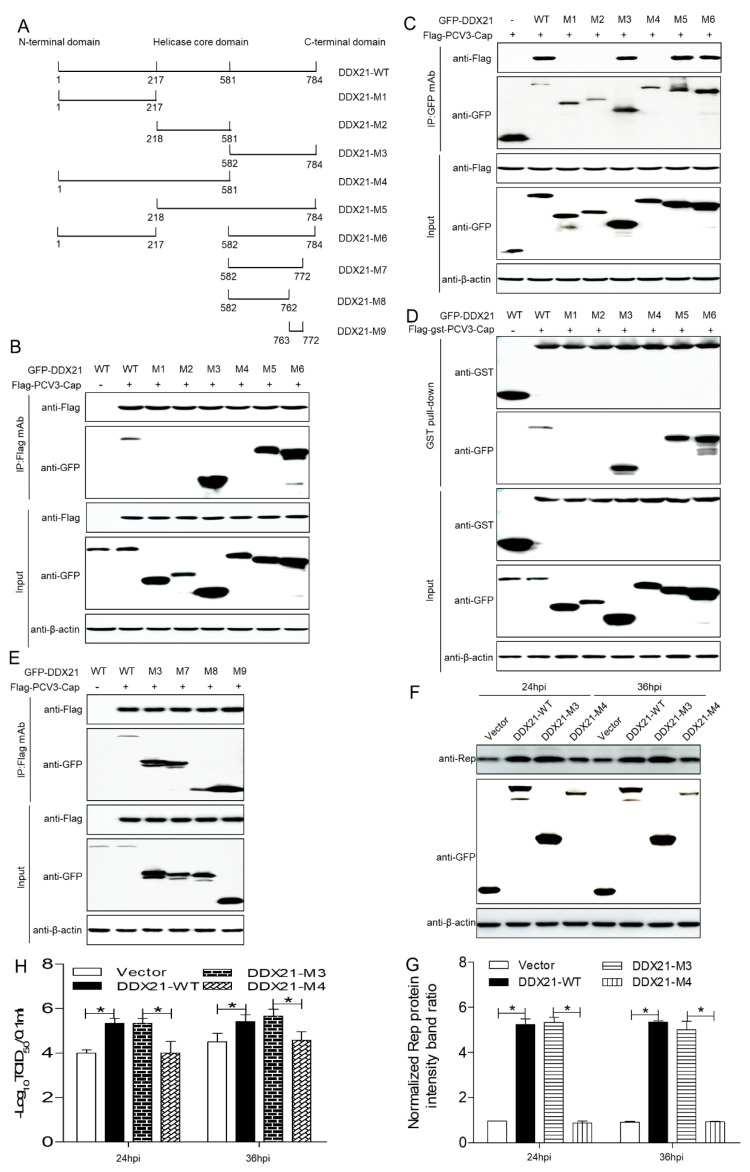
The C-terminal domain of DDX21 mediated interaction with PCV3 Cap and facilitated viral replication. (**A**) Schematic representation of three functional domains of DDX21 used in this study. (**B**–**E**) The helicase domain of DDX21-(763-772aa) bound to PCV3 Cap. HEK293T cells were co-transfected with a serial of GFP-DDX21 variants M1 to M9 with FLAG-PCV3-Cap or FLAG-GST-PCV3-Cap, and cell lysates were then subjected to immunoprecipitation or GST pull-down assays. (**F**–**H**) PK-15 cells transfected with DDX21-WT, -M3, and -M4 for 24 h were inoculated with PCV3 at a MOI of 1.0 for 24, 36 hpi. Viral protein and titers were then determined by immunoblotting with anti-GFP, anti-Rep, and anti-β-actin antibodies and TCID_50_, respectively. * *p* < 0.05.

To ascertain the domain necessary for DDX21 interaction with PCV3 Cap, a series of DDX21 variants were co-transfected with FLAG-PCV3-Cap or FLAG-GST-PCV3-Cap. The findings proved that DDX21-WT, -M3, -M5, and -M6 bound to PCV3 Cap (Figure 5A–D). A novel NLS (^763^GSRSNRFQNK^772^) was predicted in order to map the amino acids indispensable to the interaction with PCV3 Cap within DDX21-CTD. Therefore, other DDX21 variants were co-transfected with FLAG-PCV3-Cap. The results proved that DDX21-WT, -M3, -M7 or -M9 interacted with PCV3 Cap (Figure 5E). We then assessed whether DDX21-CTD promoted viral replication. PK-15 cells transfected with DDX21-WT, -M3 or -M4 were inoculated with PCV3 at an MOI of 1.0. Compared to the empty vector or DDX21-NTD group, the Rep protein levels were significantly upregulated in cells transfected with DDX21-WT or DDX21-CTD (Figure 5F,G; *p* < 0.05). In addition, the viral titers in the DDX21-WT or -CTD transfected cells were also significantly increased at the indicated times (Figure 5H; *p* < 0.05). To sum up, these results demonstrated that DDX21-CTD mediated the interaction with PCV3 Cap and the facilitated viral replication.

### 3.6. DDX21 Facilitates PCV3 Replication by Reducing IFN-β Production and ISG Expression

**Figure 6:**
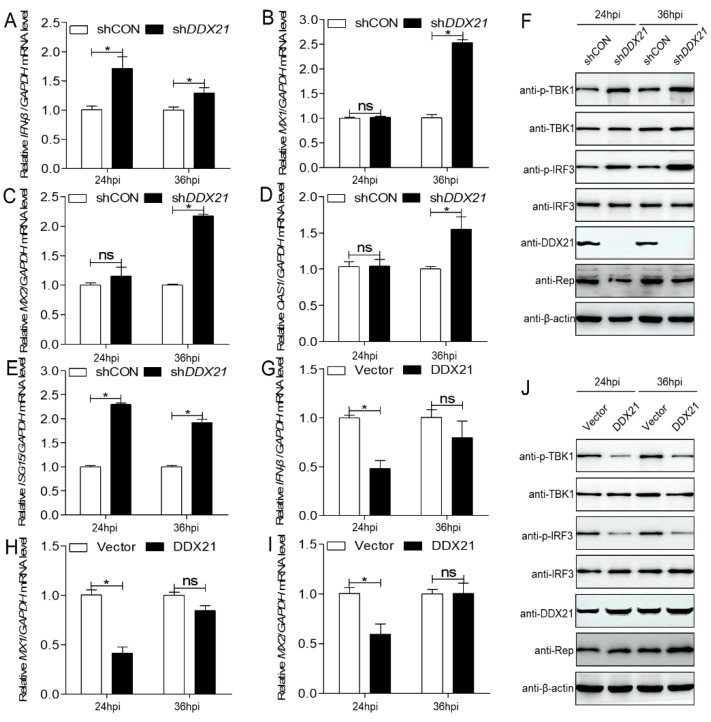
DDX21 inhibits IFN-β production and reduces ISG expression to promote PCV3 replication. (**A**–**E**) qRT-PCR was used to measure the relative *IFN-β*, *MX1*, *MX2*, *OAS1*, and *ISG15* in *DDX21*-silenced 3D4/21 cells inoculated with PCV3 at a MOI of 1.0 for 24, 36 hpi. (**F**) Immunoblotting of the proteins p-TBK1, TBK1, p-IRF3, IRF3, Rep, FLAG, and β-actin in *DDX21*-silenced 3D4/21 cells inoculated with PCV3 for 24, 36 hpi. (**G**–**I**) qRT-PCR was used to measure the relative *IFN-β*, *MX1*, *MX2*, in DDX21-overexpressing 3D4/21 cells inoculated with PCV3 at a MOI of 1.0 for the indicated time. (**J**) Immunoblotting of the proteins p-TBK1, TBK1, p-IRF3, IRF3, Rep, DDX21, and β-actin in samples from (**G**–**I**). Data are presented as mean ± SD from three independent biological experiments. ns, not significant, *p* > 0.05; * *p* < 0.05.

DDXs have antiviral activity that mainly regulates IFN-β production or interacts with viral proteins [23]. Therefore, the role of porcine *DDX21* on *IFN-β* expression was used in order to investigate the underlying pro-viral mechanism of DDX21 in PCV3-infected 3D4/21 cells at 24 and 36 hpi. The sh*DDX21* cells were inoculated with PCV3, followed by detecting the porcine *IFN-β* mRNA. *DDX21* knockdown significantly upregulated *IFN-β* mRNA at 24 and 36 hpi (Figure 6A; *p* < 0.05). The antiviral roles of IFN-β are primarily attributed to the induction of many ISGs; therefore, we further verified the role of DDX21 in modulating ISG induction during PCV3 infection. The *DDX21* knockdown upregulated the *MX1*, *MX2*, and *OAS1* mRNA expression levels at 36 hpi (Figure 6B–D; *p* < 0.05) and *ISG15* at 24 and 36 hpi (Figure 6E; *p* < 0.05) and significantly increased the p-TBK1 and p-IRF3 protein expression levels at 24 and 36 hpi (Figure 6F). DDX21 overexpression also downregulated the *IFN-β*, *MX1*, and *MX2* mRNA expression levels (Figure 6G–I; *p* < 0.05) and significantly reduced the p-TBK1 and p-IRF3 protein expression levels at 24 and 36 hpi (Figure 6J). The data demonstrated that the pro-viral activity of DDX21 is partially connected with the reduction in IFN-β production and ISG expression during PCV3 infection.

## 4. Discussion

DEAD-box RNA helicases are responsible for different RNA metabolism, as well as critical for regulating innate immune responses [34]. Many DDXs activate innate immune signaling [20,35]. However, other DDXs inhibit innate immunity [26,36]. Herein, we found that DDX21 binds directly to PCV3 Cap and reduces IFN-β and ISG production to promote PCV3 replication through shRNA-mediated *DDX21* depletion in 3D4/21 cells (Figure 6). In our previous paper [37], we used Western blotting, interaction assays, and knockdown analyses to demonstrate the interaction between DDX21 and Cap facilitating PCV2 replication in PK-15 cells. However, how the interaction between DDX21 and Cap promotes PCV2 replication remained obscure, and we had not yet found the underlying molecular mechanism.

DDX21 is involved in sensing viral RNA to activate innate immunity. The studies about the impact of DDX21 on IFN-β expression have been controversial. However, several reports have proven that the knockdown of *DDX21* inhibits the activation of the IFN-I and NF-κB pathways [20]. Other reports have proven that DDX21 overexpression enhances the activation of ISRE promoters [26]. Conversely, another report demonstrated that DDX21 overexpression did not change the IFN-β expression, but the knockdown of *DDX21* inhibited IFN-β expression [25].

DDX21 is a multifunctional enzyme with three different functional domains that participates in many RNA metabolism processes [17,38,39]. Previous studies have proven that DDX21 can interact with different types of RNA [18,40,41]. Another study demonstrated that DDX21 interacts with G-quadruplex RNA and the CTD is required for dual interaction [17]. Another study also showed that DDX21 is a dsRNA-binding protein [21]. In addition, RIG-I, MDA5, and TLR3 are the PRRs that sense dsRNA [42,43,44,45], while TLR7 and TLR8 are the major PRRs that sense ssRNA [46]. Thus, DDX21 possibly acts as an RNA/DNA sensor to compete with other PRRs, which attenuates the innate immune responses. However, this possibility requires more studies to confirm.

Herein, our proteomic results show that DDX21 interacts with the PCV3 Cap protein. In addition, DDX21 expression promotes PCV3 replication (Figure 2) by inhibiting IFN-β and ISG expression levels (Figure 6), and PCV3 infection prevented the repression of DDX21 to orchestrate its pro-viral role (Figure 1). Mechanistically, PCV3 Cap co-localized and interacted with DDX21 (Figure 3), and the PCV3 Cap NLS is vital for binding to ^763^GSRSNRFQNK^772^ of DDX21-CTD, which plays an essential role in promoting viral replication (Figure 4 and Figure 5). The data enhance our understanding of how PCV3 Cap facilitates viral replication and provide novel insight on the prevention and control of PCV3 infection.

## Figures and Tables

**Table 1:** Primers used for quantitative real-time PCR.

Gene Product	Sense Primer (5′ to 3′)	Antisense Primer (5′ to 3′)
*RT-DDX21*	TAGAGAAGCACGCTGAGCAC	GCAAGTTTCTGCCCCCTACT
*RT-IFN-β*	GTTGCCTGGGACTCCTCAAT	ACGGTTTCATTCCAGCCAGT
*RT-MX1*	GTCATCGGGGACCAGAGTTC	TCCCGGTAACTGACTTTGCC
*RT-MX2*	GTCATCGGGGACCAGAGTTC	CTCCACTTTGCGGTAGCTGA
*RT-OAS1*	GGCTGACCCCACCTACAATG	GGGACTGGGCTCTTGTTGTT
*RT-ISG15*	GGCAATGTGCTTCAGGATGG	CAGACCTCATAGGCGTTGCT
*GAPDH*	TCGGAGTGAACGGATTTGGC	TGACAAGCTTCCCGTTCTCC

## Data Availability

All data generated for this study are included in the article.
